# Ultrasmall iron‐gallic acid coordination polymer nanodots with antioxidative neuroprotection for PET/MR imaging‐guided ischemia stroke therapy

**DOI:** 10.1002/EXP.20220041

**Published:** 2023-01-17

**Authors:** Yujing Du, Yan Huo, Qi Yang, Zhihui Han, Linqian Hou, Bixiao Cui, Kevin Fan, Yongkang Qiu, Zhao Chen, Wenpeng Huang, Jie Lu, Liang Cheng, Weibo Cai, Lei Kang

**Affiliations:** ^1^ Department of Nuclear Medicine Peking University First Hospital Beijing China; ^2^ Institute of Functional Nano & Soft Materials (FUNSOM), Collaborative Innovation Center of Suzhou Nano Science and Technology Soochow University Jiangsu China; ^3^ Department of Radiology and Nuclear Medicine Xuanwu Hospital Capital Medical University Beijing China; ^4^ Departments of Radiology and Medical Physics University of Wisconsin‐Madison Wisconsin USA

**Keywords:** iron‐gallic acid coordination polymer nanodots, ischemic/reperfusion injury, reactive oxygen species scavenging

## Abstract

Oxidative stress from reactive oxygen species (ROS) is a reperfusion injury factor that can lead to cell damage and death. Here, ultrasmall iron‐gallic acid coordination polymer nanodots (Fe‐GA CPNs) were developed as antioxidative neuroprotectors for ischemia stroke therapy guided by PET/MR imaging. As proven by the electron spin resonance spectrum, the ultrasmall Fe‐GA CPNs with ultrasmall size, scavenged ROS efficiently. In vitro experiments revealed that Fe‐GA CPNs could protect cell viability after being treated with hydrogen peroxide (H_2_O_2_) and displayed the effective elimination of ROS by Fe‐GA CPNs, which subsequently restores oxidation balance. When analyzing the middle cerebral artery occlusion model, the neurologic damage displayed by PET/MR imaging revealed a distinct recovery after treatment with Fe‐GA CPNs, which was proved by 2,3,5‐triphenyl tetrazolium chloride staining. Furthermore, immunohistochemistry staining indicated that Fe‐GA CPNs inhibited apoptosis through protein kinase B (Akt) restoration, whereas western blot and immunofluorescence indicated the activation of the nuclear factor erythroid 2‐related factor 2 (Nrf2) and heme oxygenase‐1 (HO‐1) pathway following Fe‐GA CPNs application. Therefore, Fe‐GA CPNs exhibit an impressive antioxidative and neuroprotective role via redox homeostasis recovery by Akt and Nrf2/HO‐1 pathway activation, revealing its potential for clinical ischemia stroke treatment.

## Graphical abstract

Oxidative stress from reactive oxygen species (ROS) is a reperfusion injury factor that can lead to cell damage and death. Ultrasmall Fe‐GA CPNs were synthesized to exert a protective role in ischemic brain neurons via removal of ROS, rescuing of glucose metabolism, and suppressing apoptosis through the upregulation of protein kinase B (Akt), antioxidant nuclear factor erythroid 2‐related factor 2/heme oxygenase‐1 (Nrf2/HO‐1) pathway.

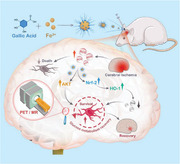

## 1 INTRODUCTION

A stroke refers to damage caused by the rupturing or blockage of blood vessels in the brain and continues to be a leading cause of lives lost, with a significant impact on healthcare expenditures across the world as well.^[^ ^1^ ^]^ A stroke can be generally categorized as ischemic (caused by a lack of blood flow) or hemorrhagic (caused by the rupturing of a blood vessel or vascular structure), with the former being more common. In the clinical treatment of patients with acute ischemic stroke, drugs are generally used to dissolve blocked arterial thrombosis, which causes thrombolysis and dredges blood vessels, improving patient recovery.^[^ ^2^ ^]^ However, thrombolysis can also cause secondary injury during reperfusion, suggesting the need for more interventions before the process of thrombolysis or following it. Since the cerebral ischemic/reperfusion (I/R) injury was firstly reported in 1968,^[^ ^3^ ^]^ more and more studies demonstrated that reperfusion injury is a potential factor of poor recovery.^[^ ^4^ ^]^ Counteracting I/R injury has attracted special attention, although the mechanism is not fully understood. Currently, oxidative stress is widely accepted as an important cause in the pathological process of I/R injury leading to cell damage and death via reactive oxygen species (ROS).^[^ ^5^ ^]^ Therefore, ROS scavengers have become a prospective candidate for I/R injury auxiliary treatment. However, difficulties in the clinical management of this injury have been reported, including different effects between genders,^[^ ^6^ ^]^ limited benefits,^[^ ^7^ ^]^ and a finite ROS‐targeting audience.^[^ ^8^ ^]^ Herein, there are immediate requirements for potent efficacy and rational safety of medicine.

Coordination polymer nanodots (CPNs), a class of burgeoning materials with self‐assembly of metals and organic ligands, have been widely used in nanomedicine because they present excellent advantages in biodegradability, biocompatibility, and loading capability.^[^ ^9^, ^10^ ^]^ Based on these benefits, significant efforts have been devoted to their application in disease diagnosis and treatment. Among them, Fe (iron) CPNs are widely utilized in the medical field. As an essential element, the introduction of Fe in Fe‐coordination polymers can reduce unnecessary toxicity concerns. Fe‐coordination polymers are also easily synthesized under inexpensive, accessible, mild, and eco‐friendly conditions.^[^ ^11^ ^]^ Furthermore, Fe‐coordination polymers provide catalytic activity and possess photothermal properties and magnetic characteristics, which can be employed in chemodynamical–photothermal synergistic therapy and magnetic resonance imaging.^[^ ^12^ ^]^ For example, CPNs with Fe^3+^ can curtail the transverse and longitudinal relaxation times of protons from bulk water, which may be employed as great T1‐weighted contrast agents. In the case of a stroke, iron is a nontoxic, biocompatible, and metabolizable metal ion, which can travel into the cytoplasm from the extracellular environment through the formation and breakage of coordination bonds that respond to a pH change caused by ischemia, indicating that Fe may be a favorable metal‐ligand in CPNs to ameliorate ischemia.

Some natural products have also received extensive attention and application in coordination polymer design. Gallic acid (GA) exerts antioxidant, antitumor, and anti‐inflammatory effects, and is widely used in nanomedicine strategies.^[^ ^13^ ^]^ Some studies suggest that GA can reduce the expression of inflammatory cytokines and elevate anti‐inflammatory cytokines, including INF‐γ, TNF‐α, interleukin (IL)‐1β, IL‐6, IL‐17, and IL‐23.^[^ ^14^ ^]^ GA also suppresses neutrophil infiltration with low toxicity to normal cells.^[^ ^15^ ^]^ A recent study also found that after GA treatment, the amelioration of inflammation was accompanied by a normalization of redox balance, as seen through glutathione (GSH)/malondialdehyde (MDA) level.^[^ ^16^ ^]^ Furthermore, while it has been reported that pure GA can exert beneficial effects on impairments following brain injury‐induced I/R, it displayed poor absorption and rapid elimination.^[^ ^17^ ^]^ Therefore, it appears that the function of GA as a ligand in CPNs may be more conducive to its biological functions. Previously, we successfully synthesized Fe‐GA CNPs (iron‐gallic acid coordination polymer nanodots) via a simple method and found that they successfully fused the characteristics of the Fe and GA, playing a synergistic role in cancer therapy,^[^ ^18^ ^]^ and indicating the potential promise of Fe‐GA CNPs as an approach for stroke treatments.

Fe‐GA CNPs were further applied as ROS scavengers to treat I/R injury monitored by PET/MR and pathological examinations. As mentioned earlier, Fe functions as a biocompatible metal‐ligand and was in a facile manner in chelating with GA. As a result, Fe‐GA CPNs scavenged ROS with low toxicity, which may ascribe to the potent antioxidant property of GA. In animal studies, neurological recovery was observed and the expected improvement of ^18^F‐FDG uptake was effectively shown by microPET and PET/MR, which may be due to the remodeling of neurons and fibers as reflected by pathology. Looking more in‐depth, we also found that Fe‐GA CNPs can promote the recovery of glucose metabolism, suppress apoptosis through the upregulation of protein kinase B (Akt) expression, stimulate antioxidant nuclear factor erythroid 2‐related factor 2/heme oxygenase‐1 (Nrf2/HO‐1) pathway, and gradually alleviate oxidation dysfunction. Our work demonstrates how Fe‐GA CNPs can effectively work in the middle cerebral artery occlusion (MCAO) model, highlighting its great efficacy in physical movement and glucose metabolism recovery as well as normalization of oxidative stress and apoptosis in ischemia stroke therapy.

## 2 RESULTS AND DISCUSSION

### 2.1 Characterization and ROS scavenging assays of Fe‐GA CPNs

**FIGURE 1:**
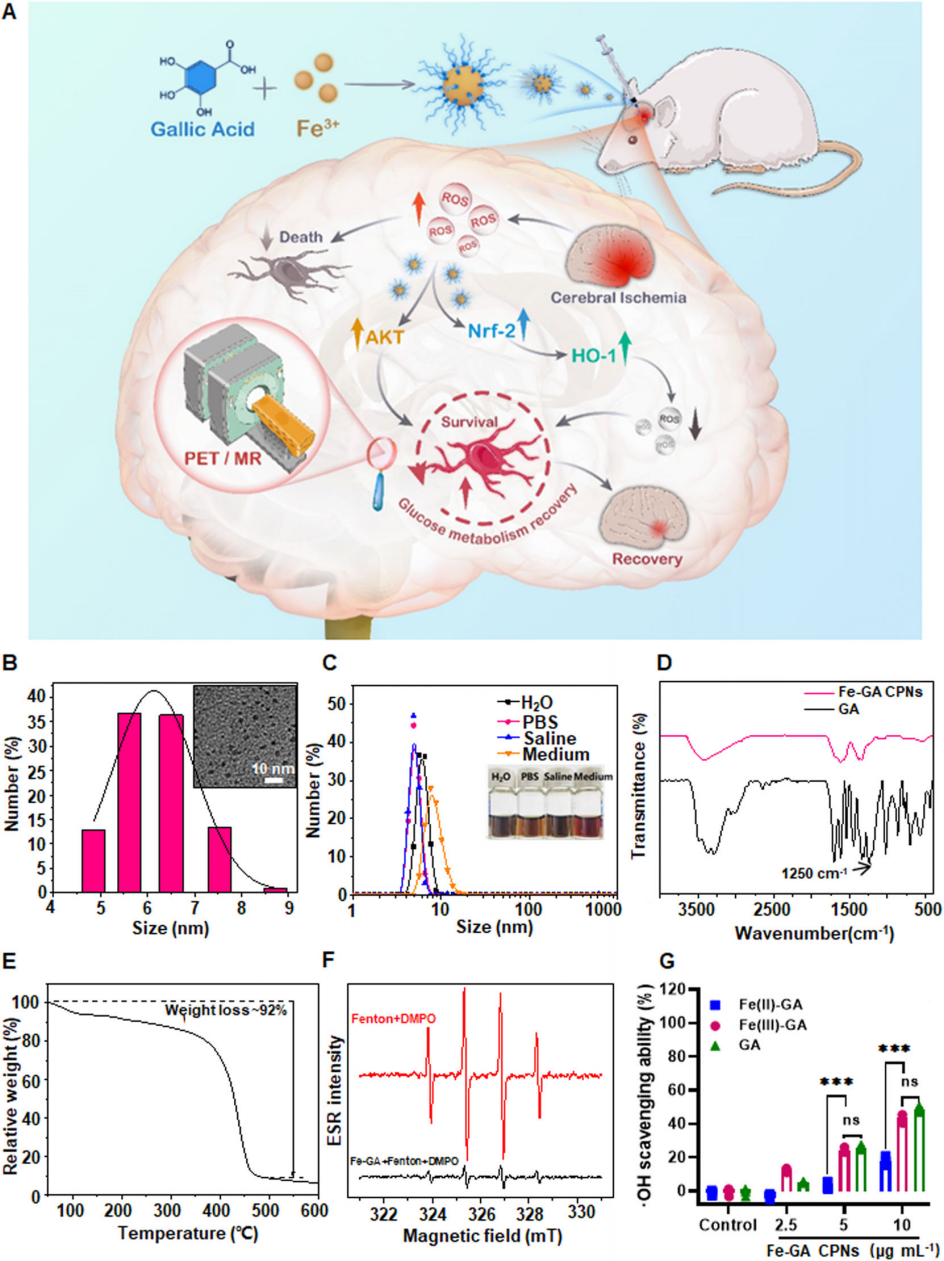
Synthesis and characterization of Fe‐GA CPNs. (A) A scheme of this study. (B) TEM images of ultra‐small Fe‐GA CPNs particles and hydrodynamic diameters (HDs). (C) UV–vis–NIR absorbance spectra in H_2_O (0.05 mg ml^−1^). Inset: A view of Fe‐GA CPNs in H_2_O, phosphate‐buffered saline (PBS), saline, and medium. (D) The transmittance of GA and Fe‐GA CPNs under different wavelengths. (E) Weight loss of Fe‐GA CPNs under various thermal parameters. (F) Characteristic peak of DMPO in the ESR spectrometer. (G) The comparation of ROS scavenging ability of GA, Fe(II)‐GA, and Fe(III)‐GA, detected by the oxTMB probe

As a proof‐of‐concept, ultrasmall Fe‐GA CPNs were synthesized in a simple, effective, low‐cost, and environmentally‐friendly manner to exert a protective role in ischemic brain neurons via removal of ROS, rescuing of glucose metabolism, and the promotion Akt and Nrf2/HO‐1 pathways (Figure 1A). TEM images indicated the excellent dispersity of relatively uniform diameter of ∼5–7 nm of Fe‐GA CPNs (Figure 1B). The size distribution was characterized by DLS analysis in different physiological solutions, including H_2_O, PBS, saline, and medium. Fe‐GA CPNs protected by polyvinyl pyrrolidone had similar diameters in the above solutions, indicating the good stability of the synthesized Fe‐GA CPNs (Figure 1C). When looking at natural GA, given its low water solubility and dispersity, it has poor bioavailability. Therefore, data implies that Fe‐GA CPNs may possess more favorable traits for biological utilization compared to natural GA. Meanwhile, fourier transform infrared (FT­IR) spectra (Figure 1D) presented that the infrared intensity (the HO–C stretching band) of the Fe‐GA particles at 1250 cm^−1^ was lower than natural GA, indicating the successful coordination of the HO–C phenolic hydroxyl group of GA with Fe^3+^.^[^ ^13^ ^]^ A change in solution color from yellow to deep‐brown also implied successful coordination between ferric ions and the phenol groups of GA.^[^ ^19^ ^]^ To determine the molar ratio of Fe^3+^ and GA, thermal gravimetric analyzer (TGA) analysis was used and the molar of Fe^3+^:GA was determined to be ∼3:1 (Figure 1E).

**FIGURE 2:**
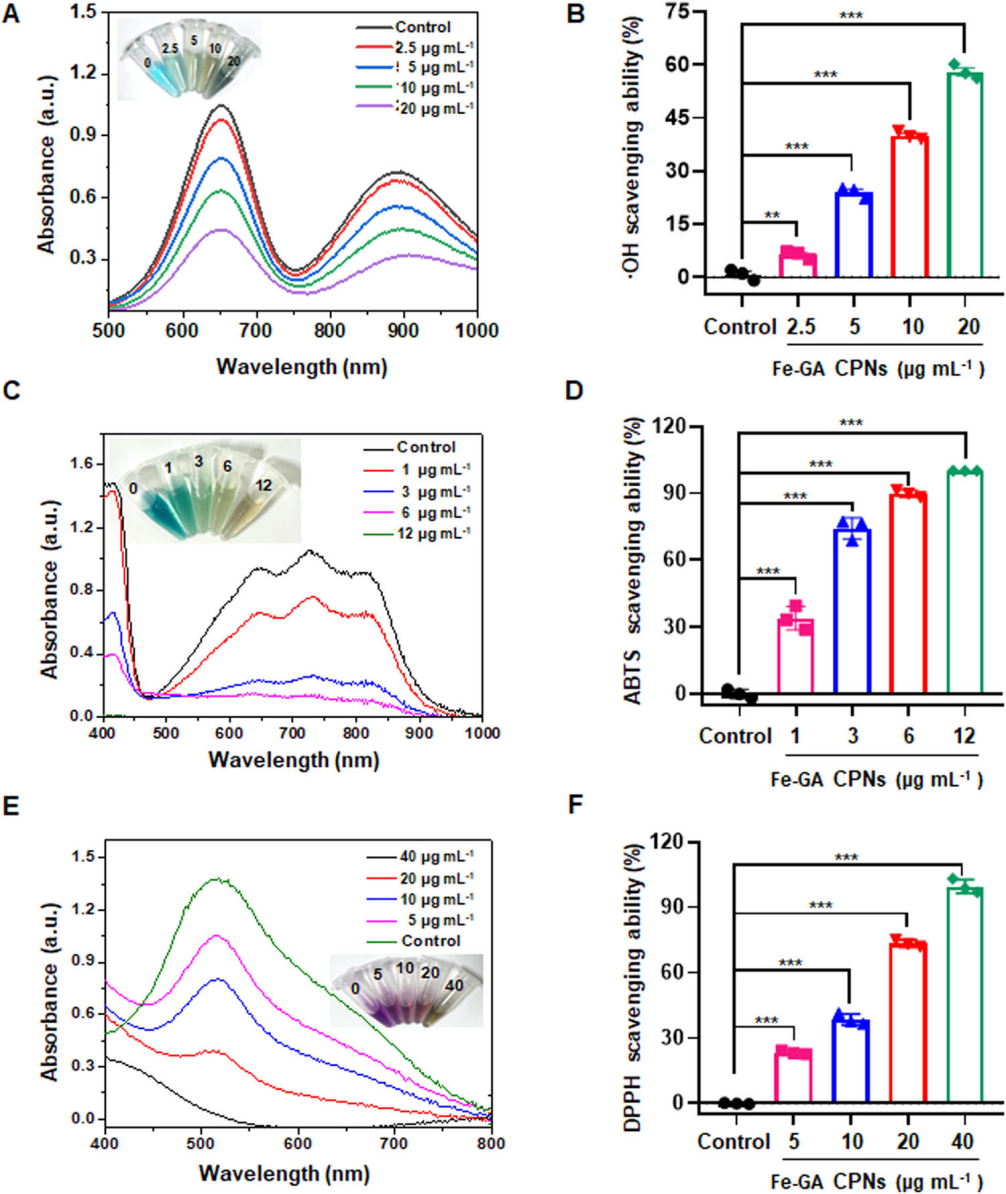
The results of ROS scavenging assays in vitro. (A,B) Fe‐GA CPNs cleared the hydroxyl free radical by measuring the decrease of MB at ∼660 nm and the quantitative analysis (*n* = 3). (C,D) Absorption of ABTS radicals at ∼734 nm and the quantitative analysis (*n* = 3). (E,F) DPPH assay at ∼517 nm and the quantitative results (*n* = 3)

Excessive hydroxyl radicals (·OH) can cause DNA damage and lipid peroxidation in the pathogenesis of ischemic stroke, indicating the importance of evaluating the antioxidant properties of Fe‐GA CPNs. The classic Fenton reaction was used by mixing Fe^2+^ and H_2_O_2_ to produce ·OH. The ·OH generated by the Fenton reaction can be detected by electron spin resonance (ESR) using 5,5‐dimethyl‐1‐pyrroline‐*N*‐oxide (DMPO), a radical trapping agent (Figure 1F). As evidenced from oxTMB probe (Figure 1G), the antioxidant capacity of Fe(II)‐GA was greatly disturbed due to the important role of ferrous ions in Fenton reaction. However, the Fe(III)‐GA and GA had a similar ROS scavenging effect, suggesting Fe^3+^ may not affect the antioxidant capacity of GA. Furthermore, it was found that the ·OH generated by the Fenton reaction was effectively scavenged by Fe‐GA CPNs as indicated by the absorption of 3,3′,5,5′‐tetramethylbenzidine (TMB). TMB would be blue and become oxTMB by ·OH, therefore, the characteristic absorption of TMB reflected the scavenging efficiency of Fe‐GA CPNs. After a sufficient response, the characteristic absorption of TMB in the control group (TMB + Fenton) showed obvious absorption characteristics, whereas the experimental group (TMB + Fenton + ∼2.5–20 μg ml^−1^ Fe‐GA CPNs) disappeared (Figure 2A,B), indicating that Fe‐GA CPNs could scavenge ·OH and prevent ·OH‐mediated oxidative damage. The Fe^3+^/Fe^2+^ system may also participate in the Fenton reaction since the role Fe‐GA CPNs play as ROS scavengers can vary based on pH value. Generally, tumors and ischemia present acidic conditions, and are conducive to the Fenton reaction, promoting the generation of ·OH.^[^ ^20^ ^]^ However, unlike tumors, the pH value of an ischemia lesion can rise with reperfusion and vascular recanalization, which reduces the production of ·OH through the Fenton reaction. According to the above MB assay results, Fe‐GA CNPs significantly reduced the production of ·OH, which suggests that it could counteract the iron‐related Fenton reaction or Fenton‐like reaction in the biological environment. Furthermore, 2,2′‐azidine‐bis‐3‐ethylbenzothiazolin‐6‐sulfonic acid (ABTS) assay and 1‐diphenyl‐2‐trinitrophenylhydrazine (DPPH) probes were used to assess free radical scavenging ability. After reacting with Fe‐GA CPNs, the characteristic absorption peaks of ABTS and DPPH at ∼734 nm and ∼517 nm decreased rapidly, and it was observed that the scavenging ability of DPPH and ABTS were correlated with Fe‐GA CPNs concentration (Figure 2C–F). The strong ROS removal ability of Fe‐GA CNPs is generally attributed to GA, given the presence of many phenol or diketone groups. Therefore, these results confirmed that Fe‐GA CPNs could be used as an antioxidant nanoplatform for effective ROS scavenging.

**FIGURE 3:**
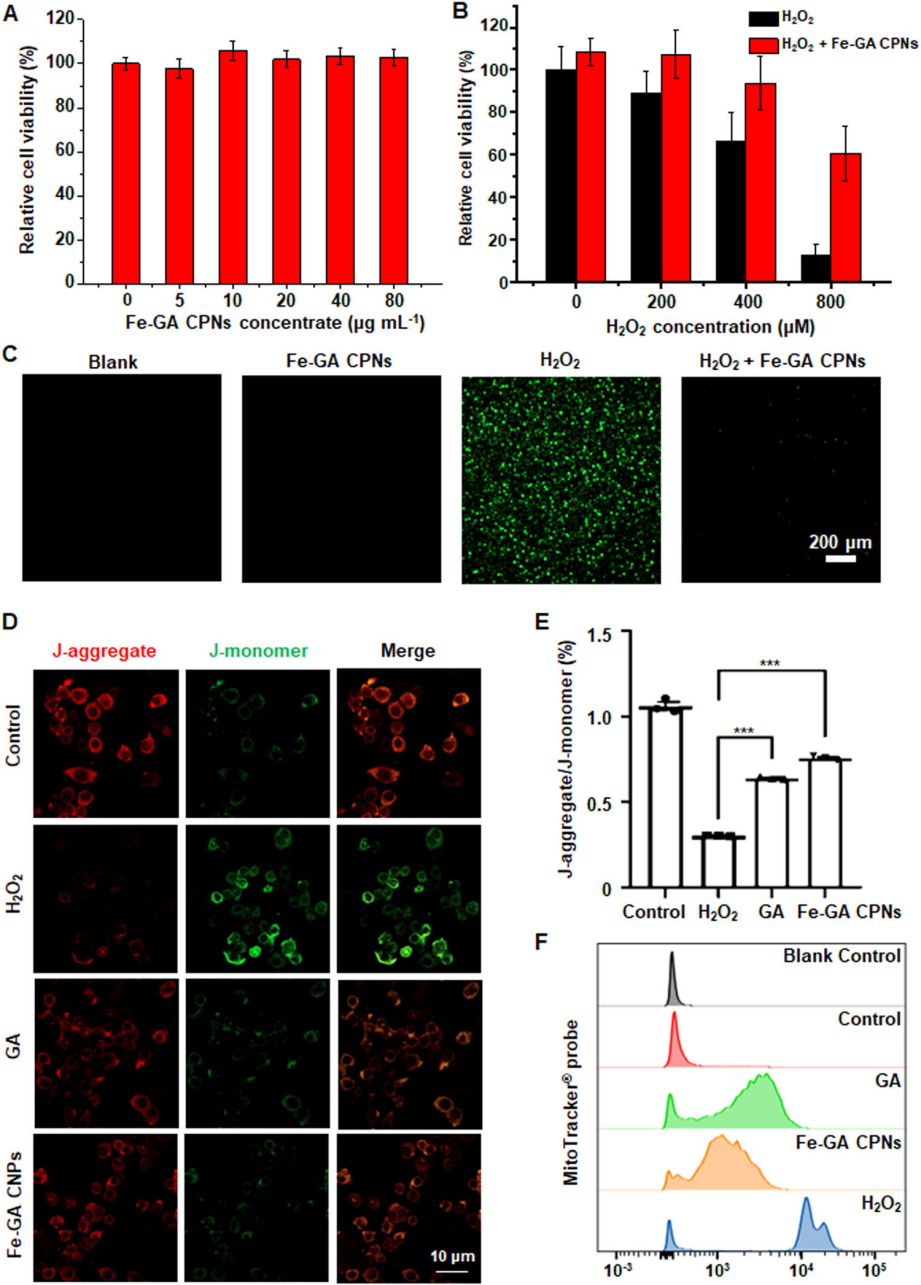
In vitro evaluation in toxicity and detoxification. (A) Relative viabilities after incubation with different concentrations of Fe‐GA CPNs. (B) The comparison of relative viabilities under different H_2_O_2_ concentrations with or without Fe‐GA CPNs. (C) Fluorescent cell imaging via DCFH‐DA. Green fluorescence indicated intracellular ROS generated by H_2_O_2_. (D) JC‐1 staining showed the fluorescence of mitochondrial transmembrane potential and depolarization. (E) Percentage of J‐aggregate/J‐monomer (*n* = 3). (F) Flow cytometry analysis of mitochondrial ROS via a mitochondrial ROS probe, MitoTracker probe (*n* = 3)

When evaluating the cytotoxicity of Fe‐GA CPNs and the protection from H_2_O_2_ stimulated cells in vitro, human umbilical vein endothelial cells (HUVEC) cells and mouse monocyte‐macrophage leukemia cells (RAW264.7) were used as a cellular modal to evaluate antioxidant property. The methyl thiazolyl tetrazolium (MTT) assay indicated that Fe‐GA CPNs had excellent biocompatibility, and there was no obvious cytotoxicity even under high concentrations (Figure 3A). Cells were then treated using different concentrations of H_2_O_2_ to induce the process of oxidative stress. Compared with the H_2_O_2_ treatment group, Fe‐GA CPNs pretreated cells showed significantly increased cell survival levels (Figure 3B). 2′,7′‐dichlorodihydrofluorescein diacetate (DCFH‐DA) was also used to verify the ability of Fe‐GA CPNs to eliminate intracellular ROS, as a fluorescent probe sensitive to oxidative stress (Figure 3C). Compared with the control group, excess ROS were generated in the cells when H_2_O_2_ was incubated with cells, whereas the level of intracellular ROS in Fe‐GA CPNs treated group were not affected. The green fluorescence intensity of the cells pretreated with Fe‐GA CPNs significantly decreased after H_2_O_2_ treatment, indicating that Fe‐GA CPNs effectively scavenged ROS to protect against ROS‐induced cell damage. JC‐1 staining and a mitochondrial ROS probe (MitoTracker probe) were also performed to exhibit the effect of excess ROS generation on mitochondria. In JC‐1 staining, intact living cells in the control group displayed a red JC‐1 aggregate signal, while the apoptotic cells in the H_2_O_2_ group showed decreased orange fluorescence and increased green JC‐1 monomer signals due to the depolarization of mitochondrial membrane potential (Figure 3D). In the H_2_O_2_ with GA/Fe‐GA CPNs group, the fluorescence of living cells was maintained, while the green fluorescence was significantly reduced, and the percentage (J‐aggregate/J‐monomer) was statistically higher compared to the H_2_O_2_ group (*p* < 0.001, Figure 3E), indicating the synergistic relationship between antioxidation and subsequent anti‐apoptosis in Fe‐GA CPNs. In MitoTracker probe staining (Figure 3F), mitochondrial ROS was significant induced by H_2_O_2_ and under GA/Fe‐GA CPNs treatment, the mean fluorescence intensity (MFI) was both sharply reduced by 75% approximately (*p* < 0.001). Of note, the MFI of Fe‐GA CPNs was also obviously lower than GA‐treated group (2475.00 ± 794.08 vs 1442.67 ± 134.56, *p* < 0.01), indicating that Fe‐GA CPNs can eliminate the production of mitochondrial ROS, or even better than GA.

### 2.2 Neurological function and imaging evaluation of Fe‐GA CPNs in vivo

**FIGURE 4:**
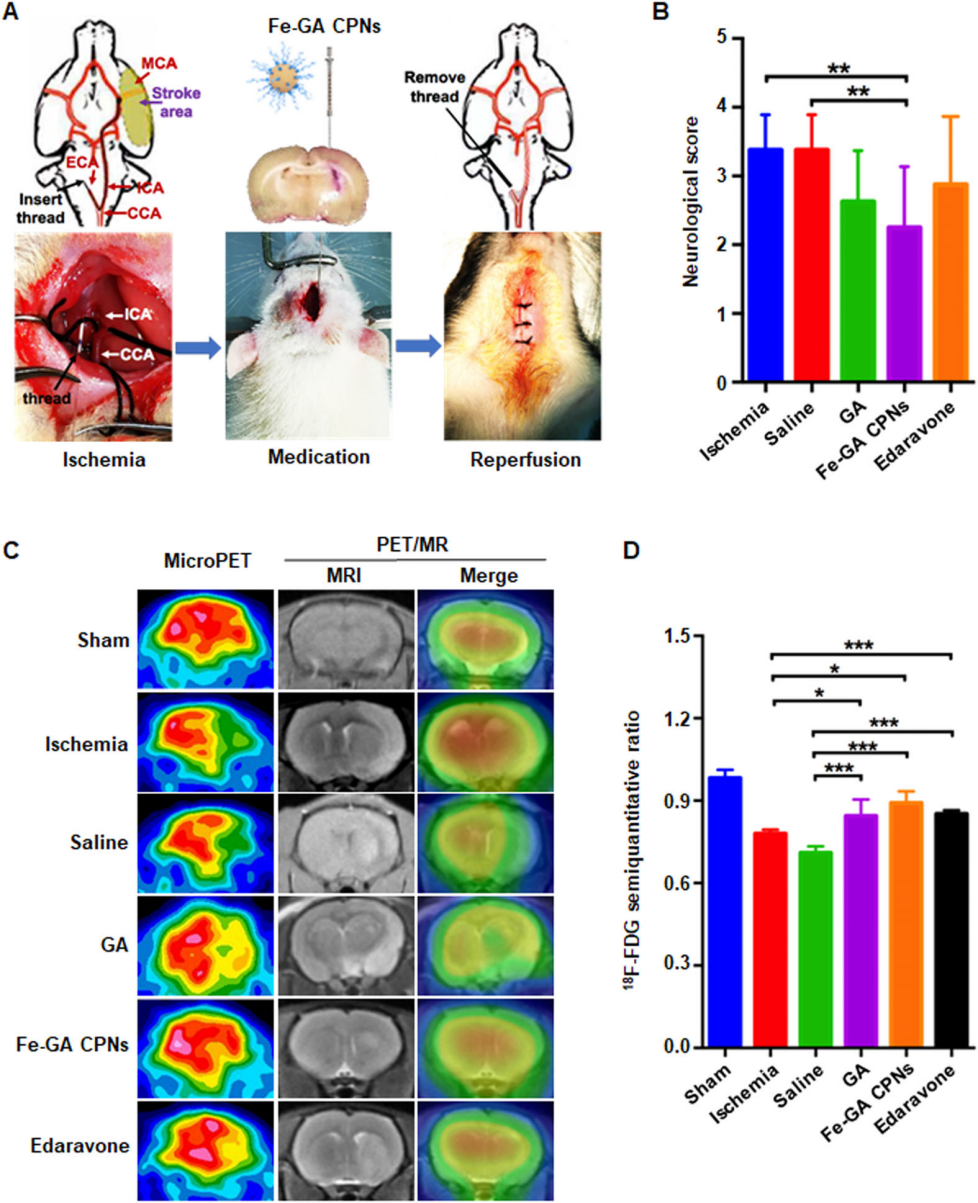
In vivo imaging evaluation in MCAO model. (A) Schematic illustration of model operation and injection workflow. (B) The results of the neurological function score (*n* = 8) indicated Fe‐GA CPNs potently reduced the infarct area. (C) MicroPET and PET/MR evaluation of rat brain after the intravenous injection of ^18^F‐FDG after treatment. (D) The uptake ratio of the ischemic brain to the normal brain was analyzed semi‐quantitatively via microPET (*n* = ∼5–8). *, **, and *** indicate *p* < 0.05, *p* < 0.01, and *p* < 0.001, respectively

Encouraged by the above previous results, we conducted MCAO models which mimic clinical progress during a stroke for further in vivo evaluation (Figure 4A). 30 min after occlusion, Fe‐GA CPNs/GA, normal saline (NS), and edaravone were administrated separately (*n* = 8). The neurological function results for each group were provided in Figure 4B. MCAO and NS groups both received a score of 3.37 ± 0.52. After treatment, the scale score of Fe‐GA CPNs, edaravone, and GA were 2.25 ± 0.89, 2.88 ± 0.89, and 2.62 ± 0.74, respectively. ANOVA analysis showed that only Fe‐GA CPNs group was significantly different from MCAO and NS groups (*p* < 0.01), suggesting that Fe‐GA CPNs exert an excellent role on the recovery of neural function. Ordinarily, infarction areas incite contralateral motor and sensory paralysis,^[^ ^21^ ^]^ with the recovery of motor sensation closely related to the plasticity of cortical projections after stroke.^[^ ^22^ ^]^ Thus, we reckoned that the outcome of Fe‐GA CPNs occurred due to the remodeling of the motor and sensory mapping and connection.

PET/MR can provide structural and functional information via one scanning and play a key role in neuroimaging. In this study, ^18^F‐FDG PET/MR and microPET were used to show the structural changes of cerebral infarction and the cerebral glucose utilization of the cerebral infarction area and cerebral blood flow.^[^ ^23^ ^]^ As illustrated in Figure 4C, there were T2‐weighted strong signals in the region of basal ganglia and temporal lobe, which indicated an infarction area present in the middle cerebral artery blood supply in both MCAO and NS groups. After treatment, the T2‐weighted strong signals were extensively decreased, particularly by Fe‐GA CPNs, in which a slightly high T2 signal was found only in the basal ganglia. It was reported that the nerve cells in the striatum would be irreversibly damaged and the dorsolateral cortex overlying the striatum and other cortex were reversible infarctions,^[^ ^24^ ^]^ which was closely aligned with the regions healed by Fe‐GA CPNs. Meanwhile, the semiquantitative ^18^F‐FDG (infarction/normal) analysis demonstrated that Fe‐GA CPNs, edaravone, and GA all have recovered glucose metabolism in varying degrees with ratio of 0.89 ± 0.04, 0.85 ± 0.01, and 0.85 ± 0.06, when compared to MCAO group (0.78 ± 0.01) and NS group (0.71 ± 0.02), *n* = 5, as shown in Figure 4D. The recovery of ^18^F‐FDG values in the influenced tissue was used as a sign of recoverable tissue,^[^ ^25^ ^]^ indicating that Fe‐GA CPNs exhibited an excellent therapeutic value.

### 2.3 Pathological and biochemical evaluation

**FIGURE 5:**
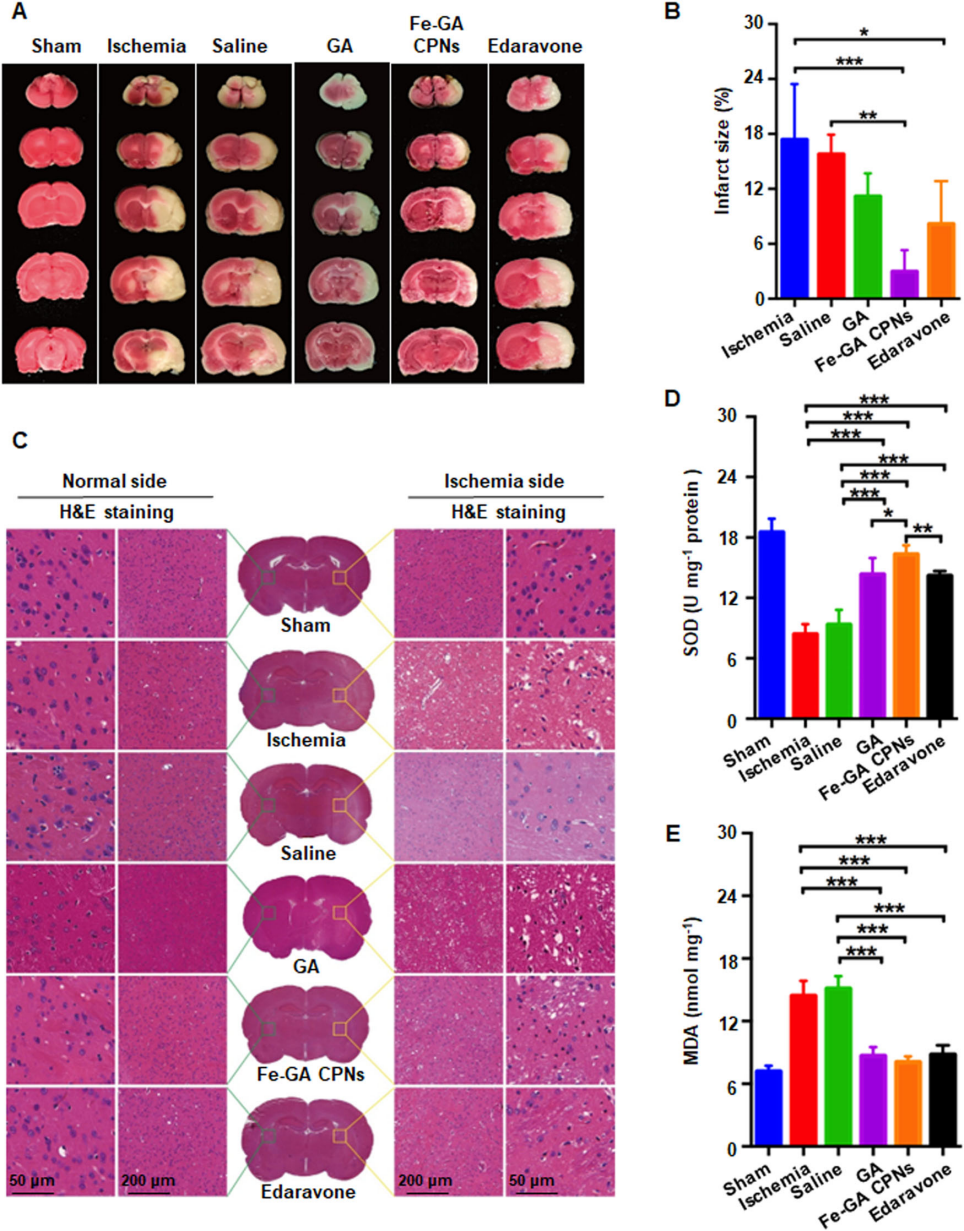
Pathological evaluation in the MCAO model. (A) Representative images of tissue slices via TTC staining. (B) TTC staining results in ischemic infarct volume (*n* = 4). (C) Representative images of brain slices via H&E staining. (D) Total SOD activity measured at 450 nm revealed Fe‐GA CPNs effectively alleviated the decrease of SOD activity (*n* = 3). (E) MDA level at 532 nm. Demonstrated Fe‐GA CPNs significantly reduce the level of lipid peroxidation stress (*n* = 3). ** and *** indicate *p* < 0.01 and *p* < 0.001, respectively

2,3,5‐triphenyl tetrazolium chloride (TTC) staining and H&E staining were utilized to evaluate the treatment effect of cerebral infarction. In Figure 5A, the infarct area in MCAO and NS groups were widely involved in the basal ganglia, frontal cortex, and temporal lobe. Following the treatment of Fe‐GA CPNs, edaravone, and GA, the injured areas decreased, which was consistent with the imaging observation. Furthermore, as evidence from TTC semiquantitative analysis, ANOVA analysis showed that Fe‐GA CPNs and edaravone obviously reduced the infarct area in contrast with MCAO or NS groups (*n* = 4). In Figure 5B, Fe‐GA CPNs, in particular, has decreased to 2.97 ± 2.36%, which reduced the MCAO or NS group by almost 90%. While GA failed to display a significant reduction. The data demonstrated that Fe‐GA CPNs exhibit more stable and significant therapeutic efficacy than GA, which may due to the improved bioavailability of GA by Fe‐based CPNs. In Figure 5C, H&E staining in the sham group revealed that nerve cell structure was intact, the boundary was distinct and the nucleolus was arranged closely and stained evenly. In contrast, in MCAO and NS groups, the neuronal nuclei were concentrated and deeply stained and the cytoplasm was crumpled and pale, which was consistent with the typical pathological manifestations of cerebral infarction. Additionally, the gap between neurons and glial cells slightly increased, suggesting early edema.^[^ ^26^ ^]^ After Fe‐GA CPNs, edaravone, or GA treatment, the morphology of neurons was improved, which was reflected in the pyknosis degree of the nucleus, tissue arrangement, and edema.

To further evaluate the ROS scavenging effect in animal studies, the measurement in situ of brain tissue of MDA, as a peroxidation product reflecting the degree of tissue peroxidation damage, and superoxide dismutase (SOD), as an antioxidant enzyme reflecting the intrinsic ability to scavenge ROS, was conducted. As shown in Figure 5D,E, in the period of cerebral ischemia‐reperfusion, ROS levels increased sharply, with MDA being deposited and SOD subsequently over‐consumed. Compared to MCAO and NS groups, the SOD activity and MDA deposition was sharply reestablished by Fe‐GA CPNs (16.32 ± 0.91 U mg^−1^, 8.06 ± 0.56 nmol mg^−1^), edaravone (14.19 ± 0.45 U mg^−1^, 8.79 ± 0.89 nmol mg^−1^), and GA (14.34 ± 1.60 U mg^−1^, 8.68 ± 0.82 nmol mg^−1^), making it close to the steady state (18.52 ± 1.35 U mg^−1^, 7.17 ± 0.55 nmol mg^−1^). Of note, the SOD activity in Fe‐GA CPNs group was even significantly higher than edaravone‐ and GA‐treated group (*n* = 3, *p* < 0.01, *p* < 0.05). Our data revealed that Fe‐GA CPNs can restore the antioxidant enzyme‐dependence pathway to a relatively normal level, reducing the burden on the antioxidant enzyme system and facilitating the transition to steady‐state, similar to previous GA‐related antioxidant treatment effects.^[^ ^27^ ^]^ After optimizing the physical and chemical properties of GA, the biological properties of Fe‐GA CPNs are well‐preserved, or to scale new heights.

### 2.4 Effect of Fe‐GA CPNs on Nrf2‐related and Akt‐associated pathway

Based on the above results, we found that Fe‐GA CPNs can alleviate pathological changes of lesions and promote neurological function by directly clearing ROS and restoring the antioxidant system. Fe‐GA CPNs also exert antioxidant and anti‐apoptotic functions through cellular signaling pathways. Nrf2 is a vital element in oxidative stress‐associated diseases, like ischemic reperfusion injury, and is physiologically expressed in the cytoplasm. Upon translocation to the nucleus, it binds to the antioxidant response element (ARE) and triggers the expression of ARE‐regulated genes, including heme oxygenase‐1 (HO‐1), an important inducible antioxidant enzyme.^[^ ^28^ ^]^ Akt is also a well‐known protein serine/threonine kinase, which is widely involved in biological regulation, such as cell survival, apoptosis, and glucose metabolism. There is a strong correlation between GA's biological activity and the Nrf2/HO‐1 pathway in previous studies ^[^ ^29^ ^]^ and Akt signaling pathway.^[^ ^30^ ^]^ Thus, we mainly evaluated the activation levels of the Nrf2/HO‐1 pathway and the Akt pathway to better evaluate the underlying mechanism of Fe‐GA CPNs.

**FIGURE 6:**
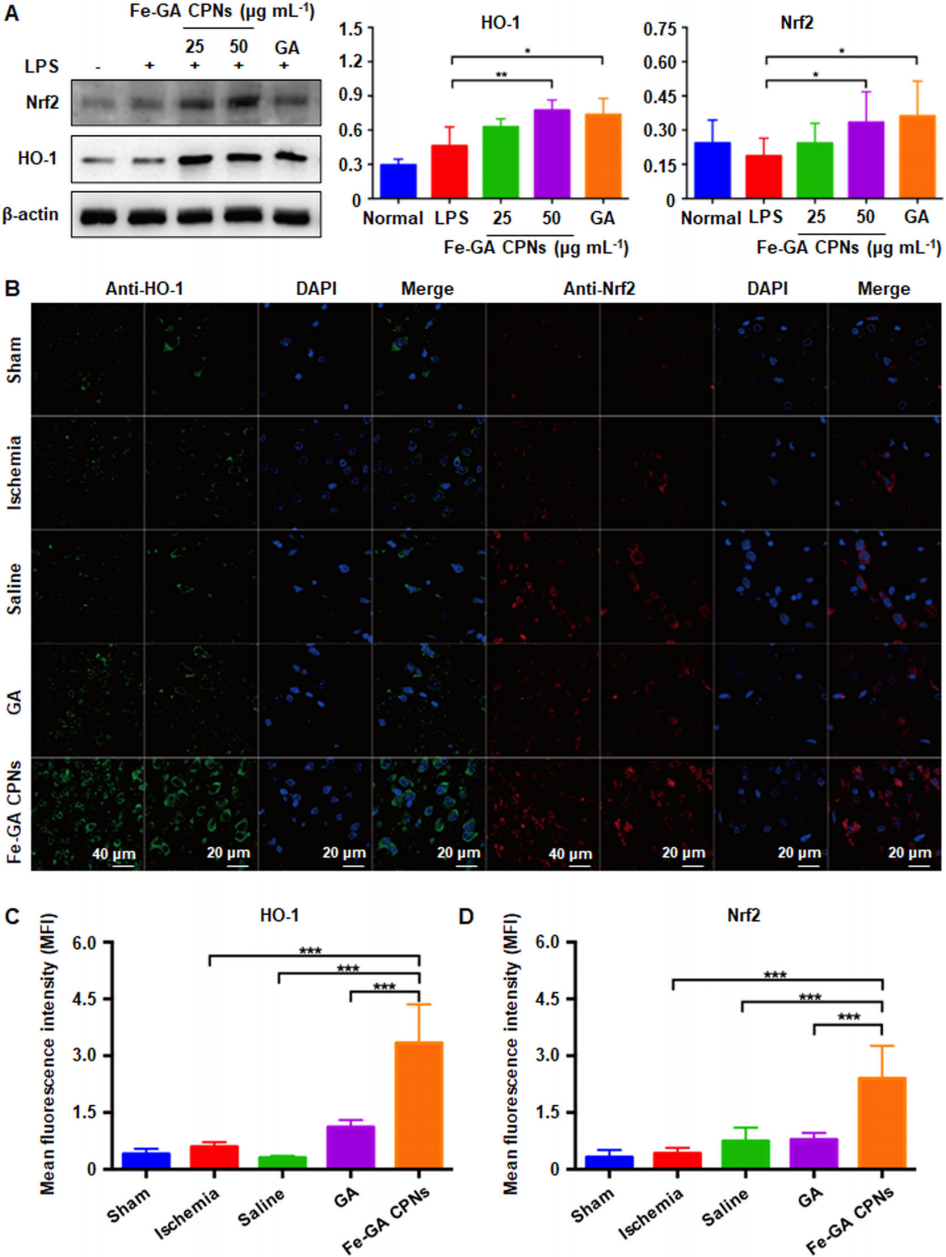
Effect of Fe‐GA CPNs on Nrf2/HO‐1 pathway. (A) Western blot and semi‐quantitative analysis showed Fe‐GA CPNs can significantly activate the Nrf2/HO‐1 pathway. (B) Immunofluorescent staining against HO‐1 and Nrf2 confirmed the strong intensity of HO‐1 in the Fe‐GA CPN treated group in brain tissue. (C) The fluorescence intensity of HO‐1 was significantly increased in the Fe‐GA CPNs group. (D) The fluorescence intensity of Nrf2 showed that Nrf2 expression was significantly upregulated by Fe‐GA CPNs treatment. *, **, and *** indicate *p* < 0.05, *p* < 0.01, and *p* < 0.001, respectively

In the LPS‐triggered ROS cell model, western blot was utilized to test the expression of Nrf2 and HO‐1 (Figure 6A). Western blot showed that the expression of Nrf2 and HO‐1 were gradually upregulated by 25 and 50 μg ml^−1^ Fe‐CPNs, as well as 50 μg ml^−1^ GA. 50 μg ml^−1^ Fe‐CPNs and GA significantly increased HO‐1 levels (*p* < 0.05) and Nrf2 expression (*p* < 0.05) compared to the LPS group. Furthermore, in the MCAO animal model, the expression of Nrf2 and HO‐1 in brain tissue was evaluated qualitatively and quantitatively using immunofluorescence staining and the nuclear translocation of Nrf2 in the damaged brain tissue (Figure 6B–D). In the sham group, the fluorescence of HO‐1 (green) and Nrf2 (red) were both weak, indicating that this signal pathway is inactivated under physiological conditions. Under GA treatment, no obvious upregulation of HO‐1 and Nrf2 were detected. However, after Fe‐GA CPNs treatment, the MFI of HO‐1 and Nrf2 were both sharply increased compared to the model group (*p* < 0.001), saline control (*p* < 0.001) and GA‐treated control (*p* < 0.001). Additionally, the nuclear translocation of Nrf2 under Fe‐GA CPNs treatment was also revealed using immunofluorescent confocal images, suggesting that Fe‐GA CPNs attenuated LPS‐ and MCAO‐induced oxidative stress damage by stimulating the Nrf2/HO‐1 pathway. Noteably, similar to the aforementioned results, the CPNs‐modified GA was more conducive to tissue uptake, thus exhibiting a more potent influence on regulating the Nrf2/HO‐1 pathway.

**FIGURE 7:**
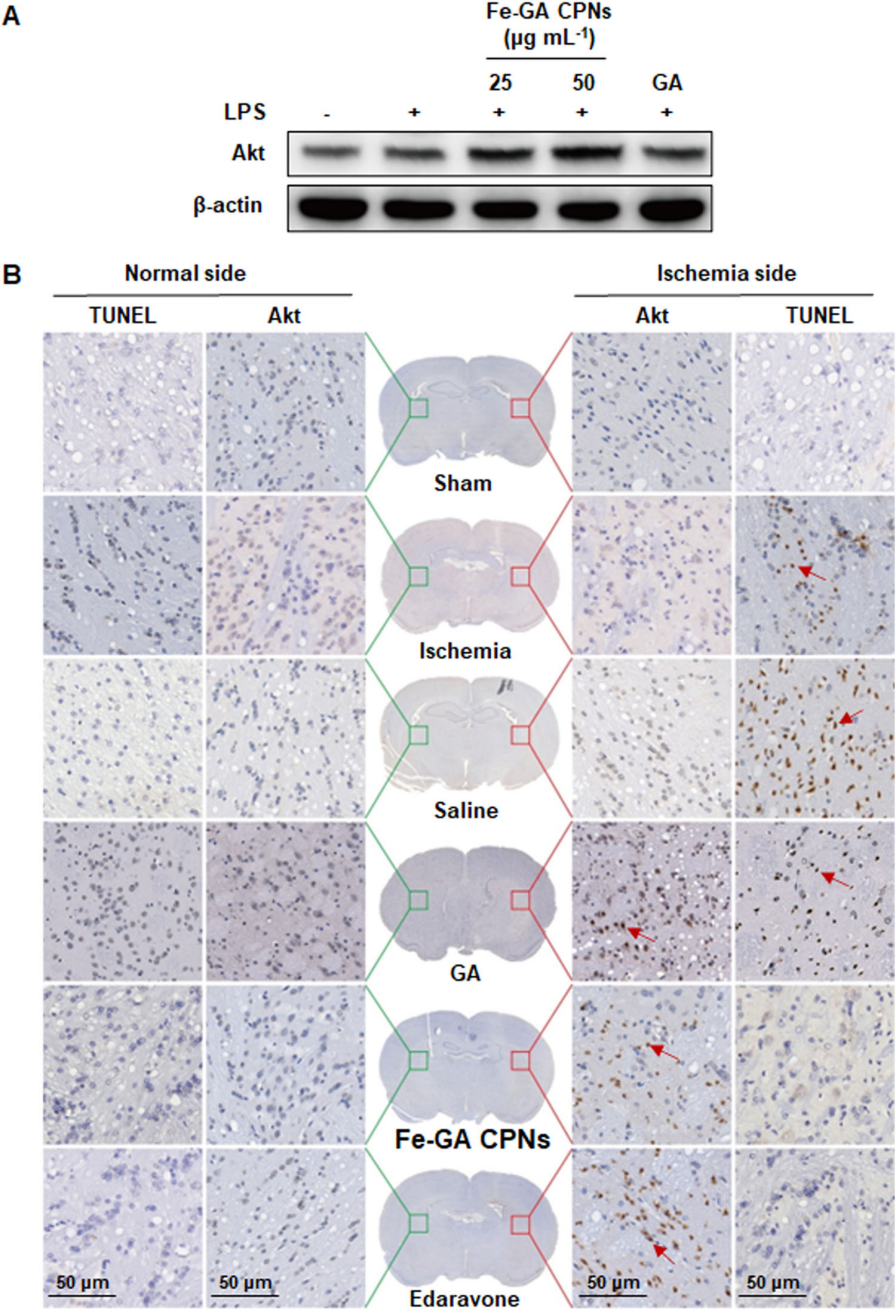
Effect of Fe‐GA CPNs on Akt and TUNEL. (A) Western blot showed that Fe‐GA CPNs can upregulate the expression of Akt, indicating that Fe‐GA CPNs may participate the Akt‐associated pathway. (B) Immunohistochemistry evaluation by TUNEL and anti‐Akt staining showed that apoptosis cells decreased and Akt expression increased in Fe‐GA CPNs treated group (as indicated by the arrows), implying that Fe‐GA CPNs can activate Akt‐related signal pathway and thereby play an anti‐apoptotic role

Western blots (WB) further confirmed that Fe‐GA CPNs can upregulate the expression of Akt (Figure 7A), especially at a concentration of 50 μg ml^−1^, which is similar to GA. In Figure 7B, the TUNEL‐positive cells (stained brown) in Fe‐GA CPNs, edaravone groups, and GA decreased to varying degrees in comparison to MCAO and NS groups. Among them, the Fe‐GA CPNs‐treated group decreased the most. Low Akt expression in the sham group was indicated by staining, and slight immunoreactivity was found in the ischemia tissue in the MCAO and NS groups. After edaravone treatment, Akt expression was significantly up‐regulated, and these results were consistent with previous studies.^[^ ^31^ ^]^ Similarly, Akt expression was also obviously up‐regulated in the Fe‐GA CPNs group and partly expressed in the GA group, due to the reestablishment of Akt expression as reported.^[^ ^32^ ^]^ Additionally, the expressed region of Akt in various treatment groups both translocated from the cytoplasm to the nucleus, suggesting Akt activation. Based on the results of TUNEL and Akt staining, the Fe‐GA CPNs‐associated mechanism was associated with an anti‐apoptotic effect relating to the activation of Akt signaling. Some studies have shown that the activation of Akt signaling (such as PI3K/Akt/mTOR pathway) can downregulate the Bax protein (a pro‐apoptotic protein) and upregulate the Bcl‐2 protein (an anti‐apoptotic protein),^[^ ^33^ ^]^ which may be one of the Fe‐GA CPNs‐associated mechanisms. Additionally, Akt acts as an important signaling pathway element and can not only participate in the anti‐apoptotic pathway but also improve glucose metabolism. ^18^F‐FDG uptake is closely associated with glucose transporters (GLUT) on the cell membrane, and GLUT1 can be closely regulated by the Akt pathway,^[^ ^34^ ^]^ which indicates that glucose recovery may be another Fe‐GA CPNs‐associated mechanism. It also has been reported that the uptake of ^18^F‐FDG can monitor the PI3K/Akt/mTOR pathway targeting therapeutic response,^[^ ^35^ ^]^ providing the imaging basis for PET/MR.

### 2.5 Biosafety of Fe‐GA CPNs

**FIGURE 8:**
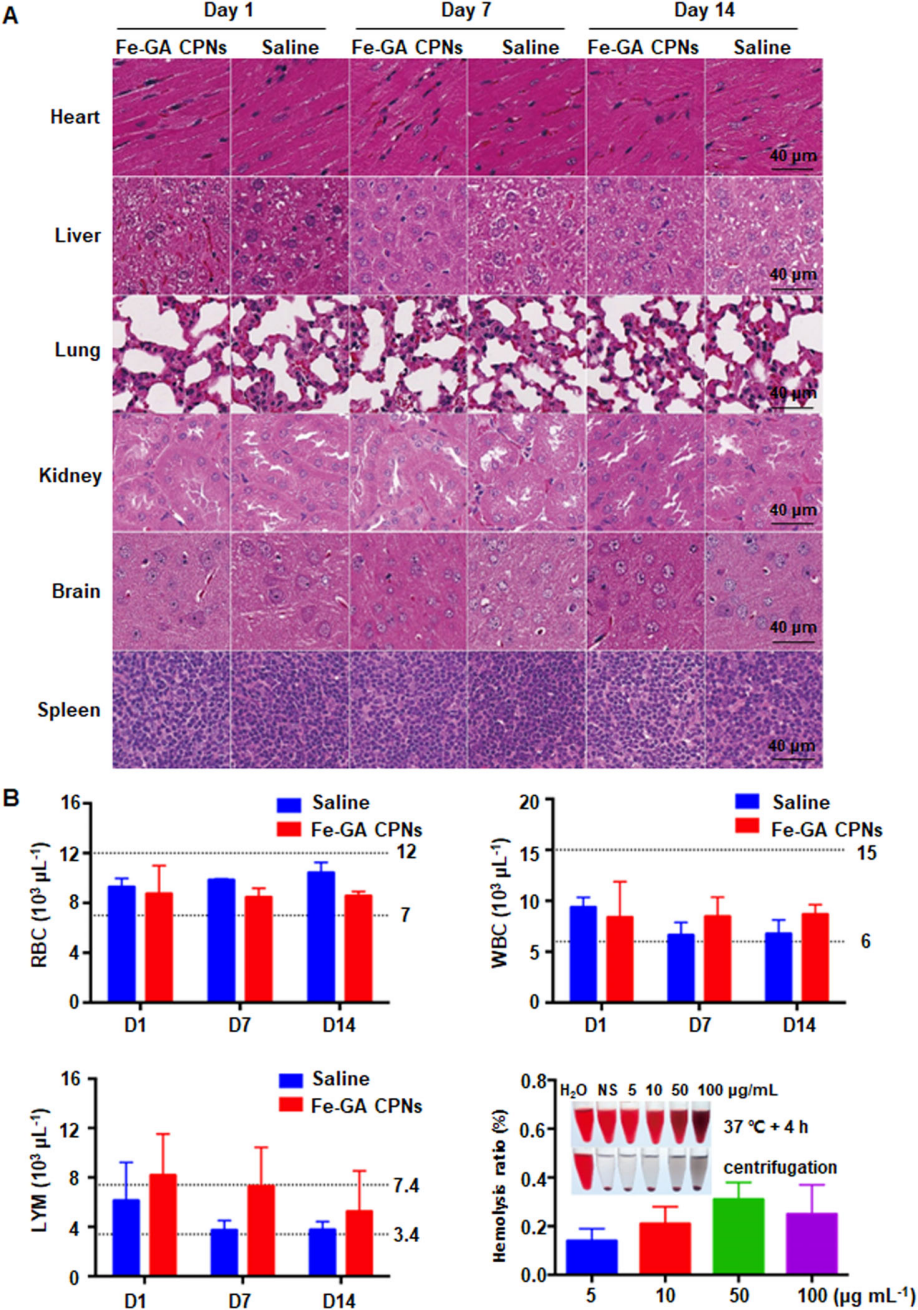
In vivo evaluation of biosafety. (A) H&E staining revealed no obvious abnormality in major organs at 1, 7, and 14 days after the intravenous injection of Fe‐GA CPNs and saline (*n* = 3). (B) The results of RBC, WBC, LYM counts, and hemolysis rates showed no significantly abnormal changes indicating the in vivo biosafety in a long‐term immune reaction and membrane tolerance (*n* = 3)

Biosafety is a key factor for new materials concerning in vivo application,^[^ ^36^ ^]^ especially when used in the brain. In this study, H&E staining and hematological tests were conducted at 1, 7, and 14 days after tail vein injection of Fe‐GA CPNs, and a hemolysis test was used to evaluate membrane tolerance (Figure 8). In Figure 8A, the structures of major organs were complete and arranged orderly, such as cardiac fibers, hepatic lobules, alveoli, renal tubules, cutaneous medullary junctions, and the white pulps of the spleen, and no obvious edema or inflammatory cell infiltration was present in the tissue. The nuclei of the tissue were full, the nucleoplasm was evenly distributed, and the cytoplasm was stained. In Figure 8B, red blood cells (RBC), white blood cells (WBC), and lymphocytes (LYM) were measured, and a hemolysis test was administered. By comparing results from the Fe‐GA CPN‐administered group to normal values and the control, a slightly minor increase in lymphocyte count was shown, but this number soon reached normal levels in later stages, indicating no significant long‐term immune reaction. Hemolysis rates in 5–100 μg ml^−1^ Fe‐GA CPNs groups also presented good erythrocyte membrane tolerance, which was lower than 1%. Our data demonstrated that Fe‐GA CPNs have good biocompatibility, and their toxicity to organisms is also negligible.

## 3 CONCLUSION

In this study, Fe‐GA CPNs were conveniently synthesized with favorable biocompatibility, and their application in stroke therapy was reported given their efficacy as a potent ROS scavenger. The following mechanisms make Fe‐GA CPNs such a promising nano‐antioxidant drug: (1) In vitro results demonstrated the ROS‐eliminating capability of Fe‐GA CPNs through the exhibition of ROS, including H_2_O_2_ and ·OH. (2) Fe‐GA CPNs can re‐establish the redox homeostasis of the antioxidant enzyme system as determined by MDA and SOD levels. (3) Fe‐GA CPNs can activate the Nrf2/HO‐1 and Akt pathways to facilitate the regulation of antioxidant activity. Surpassing GA, Fe‐GA CPNs also provide a satisfactory neuroprotective effect with improved cellular viability, reparation of neurological function and glucose metabolism, and apoptosis reduction being observed through cellular viability assays, functional scales, PET/MR imaging, and pathological examinations. Moreover, the biosafety evaluation revealed the negligible toxicity of Fe‐GA CPNs, and they also effectively optimize the physical and chemical properties of GA while still exerting their corresponding chemical and biological activities. In perspective, the excellent therapeutic potential of Fe‐GA CPNs can be applied to the treatment of stroke.

## 4 EXPERIMENTAL SECTION

### 4.1 Synthesis of Fe‐GA CPNs

Fe‐GA coordination polymer nanodots (Fe‐GA CPNs) were successfully synthesized based on our previous study.^[^ ^18^ ^]^ Briefly, the FeCl_3_ solution (100 mg ml^−1^) was added to a polyvinyl pyrrolidone (PVP) solution (100 mg PVP in 10 mL water) for 1 h. Then GA solution (10 mg ml^−1^) was added and stirred overnight. The CPN solution was dialyzed in deionized water (MWCO = 14800) for 24 h to remove excess Fe^3+^ and stored at 4°C.

### 4.2 Characterization

Images were acquired using a FEI Tecnai F20 transmission electron microscope (TEM). Malvern Nano ZS90 was used to record the dynamic light scattering (DLS) of Fe‐GA CPNs. Infrared spectroscopy was performed by the BRUKER VERTX 70 (400–4000 cm^−1^). A thermal gravimetric analyzer and inductively coupled plasma atomic emission spectrometer (ICP‐OES) were used to calculate the concentration of Fe^3+^ in Fe‐GA CPNs.

### 4.3 Cell culture

We used human umbilical vein endothelial cells (HUVEC), mouse monocyte‐macrophage leukemia cells (RAW264.7) and mouse hippocampal neuron cells (HT22), both obtained from the American Type Culture Collection (ATCC). Cell culturing followed the recommendations from ATCC. The medium was a high‐sugar medium containing 10% fetal bovine serum (FBS) and 1% penicillin/streptomycin with 5% CO_2_ at 37°C.

### 4.4 Cytotoxicity evaluation

Fe‐GA CPNs were incubated with the cells for 12 h at 0, 5, 10, 20, 40, and 80 μg ml^−1^, and the relative survival rate of cells was tested using MTT.

### 4.5 ROS scavenging assays

To analyze ROS scavenging ability, HUVEC cells were incubated with Fe‐GA CPNs (with CFe = 50 μg ml^−1^) for 12 h and incubated with H_2_O_2_ (0, 200, 400, 800 μm) for 2 h. Subsequently, 20 μm 2′,7′‐dichlorofluorescein diacetate (DCFH‐DA) was used for the detection of intracellular ROS and a confocal microscope (Zeiss LSM 800) was used to observe cells.

### 4.6 OH• scavenging assay

For 5,5‐dimethyl‐1‐pyrroline‐*N*‐oxide (DMPO) assay, DMPO is used as a trapping agent for ·OH. DMPO with Fe‐GA CPNs, H_2_O_2,_ and Fe^2+^ were mixed. After 30 s, the characteristic peak of DMPO that captured hydroxyl radicals was measured by the ESR spectrometer (JEOL Corporation JES‐X320).

### 4.7 Antioxidant capacity in vitro

For 1,1‐diphenyl‐2‐trinitrophenylhydrazine (DPPH) assay, various concentrations (0, 5, 10, 20, and 40 μg ml^−1^) of Fe‐GA CPNs and 125 μm DPPH ethanol were mixed with equal volume, and the absorption of DPPH at 517 nm was detected after 30 min. Scavenging efficiency (%) = ((A_DPPH_—A_sample_)/A_DPPH_) × 100%. A_DPPH_ is the absorption of a pure DPPH solution. A_sample_ is the absorption of the solution after adding Fe‐GA CPNs. The 2,2′‐azidine‐bis‐3‐ethylbenzothiazolin‐6‐sulfonic acid (ABTS) experiment was performed to show antioxidant capacity. We activated 7.45 mm potassium persulfate with 7.45 mm ABTS overnight. Fe‐GA CPNs (concentration = 0, 1, 3, 6, and 12 μg ml^−1^) were then mixed with ABTS radicals. The absorption of ABTS radicals was tested at 734 nm. ABTS scavenging efficiency (%) = ((A_ABTS_—A_sample_)/A_ABTS_) × 100%. A_ABTS_ is the absorption of pure ABTS free radical solution. A_sample_ is the absorption of the solution after adding Fe‐GA CPNs.

Similarly, the 3,3′,5,5′‐tetradine (TMB) probe was applied to compare the roles of Fe^3+^ and Fe^2+^ in Fe‐GA CPNs and test the scavenging effect of Fe‐GA CPNs on ·OH. We configured the TMB probe (4.8 mg, 250 μl DMSO) and obtained the oxTMB reagent by blending it with 100 μl H_2_O_2_ (10 mm) and 100 μl FeCl_2_ (2 mg ml^−1^). Fe(III)‐GA CPNs, Fe(II)‐GA CPNs and GA (concentration = 2.5, 5, 10, 20 μg ml^−1^) or Fe‐GA CPNs (concentration = 0, 2.5, 5, 10, 20 μg ml^−1^) were then mixed with oxTMB. The absorption of oxTMB was tested at 640 nm. ·OH scavenging efficiency (%) = ((A_oxTMB_—A_sample_)/ A_oxTMB_) × 100%. A_oxTMB_ is the absorption of pure oxTMB solution. A_sample_ is the absorption of the solution after drug administration.

### 4.8 Mitochondria‐associated Staining

JC‐1 staining was performed for the measurement of mitochondrial membrane potential. Fe‐GA CPNs or GA (50 μg ml^−1^) were incubated with RAW264.7 cells for 12 h and mixed with H_2_O_2_ for 12 h. Untreated cells were used as a negative control. After washing with PBS, cells were incubated for 20 min in the dark with JC‐1 dye (2 μm) at 37°C. Then, cells were washed with PBS and detected by a confocal microscope (Zeiss LSM 800).

MitoTracker probe was used to trace the generation of mitochondrial ROS. 50 μg ml^−1^ Fe‐GA CPNs/GA were incubated with HUVEC cells for 12 h and mixed with H_2_O_2_ for 2 h. HUVEC cells without MitoTracker dye was blank control group and HUVEC cells with dye was control group. All cells were gently collected and resuspended in 200 μl PBS and a 1 μl MitoTracker probe was added in each tube. After incubation for 30 min, a flow cytometer (BD Fortessa, USA) was used for the measurement with a total of 10,000 gated cells.

### 4.9 Animals

Adult Sprague‐Dawley male rats (280–300 g) were randomly assigned to five groups: the sham‐operated control group, the MCAO group, the Fe‐GA CPNs injection group, the normal saline (NS) group, the edaravone injection group, and GA injection group. The injection of Fe‐GA CPNs and GA was at a ratio of Fe: GA = 1:2. After reperfusion for 24 h, all rats underwent neurologic testing followed by a neurologic function scale to evaluate the stability and homogeneity of MCAO models, respectively. For the biosafety assessment of Fe‐GA CPNs, 4‐week‐old male ICR mice (30–40 g) were intravenously injected with Fe‐GA CPNs (100 μl, 200 μg ml^−1^) at 1, 7, and 14 days. Saline was used as a control.

### 4.10 Hematological analysis

We performed blood tests following our previous procedure.^[^ ^37^ ^]^ Briefly, mice were euthanized and whole blood samples were collected 1, 7, and 14 days after injection. Subsequently, samples were analyzed by a VetScan HM5 hematology analyzer (Abaxis). The counts of red blood cells (RBC), white blood cells (WBC), and lymphocytes (LYM) were recorded.

### 4.11 Hemolysis analysis

Fresh mouse blood was gently collected and washed with NS. Approximately 1.0 × 10^7^ cells per tube were used for hemolysis assays. 5, 10, 50, and 100 μg ml^−1^ of the Fe‐GA CPNs were incubated with the RBCs at 37°C for 4 h. Water‐ and NS‐treated groups were used as positive and negative controls, respectively. Considering the dark color of Fe‐GA CPNs, the tube with free RBCs contained the same concentration of Fe‐GA CPNs as the blank control. In each group, samples were centrifuged for 15 min at 1000 rpm, and supernatants were determined at 540 nm using a hybrid reader (Synergy H1, Bio‐Tek). The hemolysis rate was calculated as reported.^[^ ^38^ ^]^

### 4.12 Rat stroke model

The MCAO model was performed following our previous study with slight modifications.^[^ ^39^ ^]^ Anesthetization of rats was induced by inhaling 2–5% isoflurane and maintained with 2% isoflurane in a supine position. After sterilization and incision, the right common carotid artery (CCA), internal carotid artery (ICA), and external carotid artery (ECA) were exposed. Through the ECA, a silicon‐coated filament was gently inserted into the ICA until mild resistance was felt. 90 minutes later, reperfusion was performed by withdrawing the, immediately fastening the free end of the ECA, and removing the slipknot of the CCA. For intraventricular injection, animals are treated via intracerebral injection of Fe‐GA CPNs or NS or intraperitoneal injection of edaravone 30 min after finishing the occlusion. Sham rats were subjected to the same procedure but were not sutured.

### 4.13 Intraventricular injection

Intraperitoneal injection was performed using stereotaxic surgery. Rats were gently fixed with the stereotaxic device, Stereotax (Stoelting), in a symmetrical horizontal plane position. Anterior fontanelle was exposed and set as the coordinate origin. According to the spatial coordinates of the lateral ventricle, the injection position (horizontally backward 1 mm and 2.4 mm to the right) was vertically drilled. Next, at a rate of 2 μl per minute, 10 μl of Fe‐GA CPNs or NS were slowly injected through the hole to a depth of 4.0 mm beneath the durometer's surface. Subsequently, reflux was prevented by slowly removing the needle within 5 min. Finally, the wound was stitched and cleaned up. Rats were monitored during this process.

### 4.14 Neurologic function scale

Assessment of neurological functions was performed after MCAO for 24 h from a previous experiment.^[^ ^40^ ^]^ Scoring was used as follows: (1) no neurological symptoms scored 0; (2) contralateral forelimb flexion scored 1; (3) reduced grip of the right forelimb scored 2; (4) when held by the tail, the torso turns to the right scored 3 and; (5) spontaneous contralateral circling scored 4.

### 4.15 ^18^F‐FDG microPET

Approximately 18.5MBq of ^18^F‐FDG were injected via the tail vein after 24 h of reperfusion. The rats were anesthetized as mentioned above and positioned prone in the micro‐PET scanner bed 30 min after the ^18^F‐FDG injection. A 10‐min‐long static acquisition was performed, and the images were reconstructed using a MOSAIC HP PET system (Philips Corp.). This experiment used the following scan parameters: a PET axial field of view of 12.7 cm, an acquisition field of view of 12.37 cm by 12.66 cm, a maximum effective pixel area of 4096 by 4032 pixels, and a scan field of 10 cm by 9.7 cm.

### 4.16 ^18^F‐FDG PET/MR scanning

The rats were anesthetized and scanned using a PET/MR scanner (GE Signa, Hybrid TOF PET/MR) with a rat head surface coil (BioSpec 70/30 USR, Bruker). The following T2‐weighted imaging criteria were used for scanning; a triplicate imaging sequence ensured reproducible positioning of the animals in the scanner. The following T2‐weighted imaging criteria were used: repetition time = 5000 ms, echo time = 68 ms, field of view = 5.0 × 5.0 cm, matrix: 192 × 192, slice thickness = 1.5 mm, spacing = 0.5 mm. SPM 8 software (https://www.fil.ion.ucl.ac.uk/spm/) was used for pretreatment. The pre‐processing of rs‐fMRI includes time slice correction, head motion correction, and standardization.

### 4.17 2,3,5‐triphenyl tetrazolium chloride (TTC) staining

Whole brains were immediately harvested and preserved at −80°C for 10 min after scoring and then sliced into 2‐mm coronal sections. Slices were incubated at 37°C for 30 min in a 2% TTC solution. An Image‐Pro Plus 6.0 program (Media Cybernetics Company, Commerce, GA, USA) was used to quantify the infarct area (unstained). Total infarct area percentage (%) = total infarct area / total cerebral area × 100%.^[^ ^41^ ^]^

### 4.18 Hematoxylin and eosin (H&E) and immunohistology staining

Following previous procedures, H&E and immunobiological staining were performed.^[^ ^36^ ^]^ The brain, heart, lung, liver, kidney, and spleen were stained with H&E after being fixed in 4% paraformaldehyde, embedded in paraffin, and sectioned at 3 μm. In addition, slides were deparaffinized and rehydrated, and the antigen was activated by heating. The sections were incubated with the primary antibodies (anti‐AKT antibody, 1:200, Servicebio) overnight. 3,3′‐diaminobenzidine (DAB, Servicebio) was then applied as a substrate to reveal the antigen. Sections were photographed with a microscope (Nikon, Japan).

### 4.19 TUNEL staining

Using terminal deoxynucleotidyl transferase‐mediated deoxyuridine triphosphate nick‐end labeling (TUNEL) staining, apoptosis was evaluated (ZK‐8005, Zhongshan Goldenbridge Biotechnology, China) according to a standard protocol.^[^ ^42^ ^]^ The prepared sections were covered entirely with 3% H_2_O_2_ for 15 min at room temperature and then washed in PBS (5 min each, 3 times). After covering them with 5% Triton‐X100 for 10 min, they were incubated for 1 h at 37°C in a humidified atmosphere with a TUNEL reaction mixture. A light microscope was used to observe the samples after they were spotted with DAB fluid and hematoxylin.

### 4.20 Oxidative stress detection

To indicate oxidative stress, an MDA assay kit (S0131, Beyotime, China) and a total SOD assay kit (S0101, Beyotime, China) were used to measure malondialdehyde (MDA) formation and SOD activity in normal and ischemia cerebral tissues. Tissue and protein preparation was conducted by assay kit instructions. The reaction was determined by a multimode reader at 532 nm (MDA) or 450 nm (SOD). Results are reported as nmol of MDA per mg protein or expressed as U mg^−1^ protein for SOD.

### 4.21 Western blot

HT22 cells were incubated with saline, 25, 50 μg ml^−1^ Fe‐GA CPNs and 50 μg ml^−1^ GA for 12 h at 37°C. After replacing the medium with fresh medium, 100 μg ml^−1^ LPS was added for 2 h at 37°C. The total protein lysates were collected using radioimmunoprecipitation assay (RIPA) buffer (Lablead, China). Protein samples were electrophorized and transferred. The membrane was incubated overnight at 4°C with primary antibodies after blocking for 1 h, using mouse anti‐𝛽‐actin (1:2000, Santa Cruz Biotechnology, USA), rabbit anti‐Nrf2 (1:1000, ABmart, China), rabbit anti‐HO‐1 (1:2000, ABclonal, China) and rabbit anti‐Akt (1:1000, ABmart, China) overnight at 4°C. Membranes were then used for secondary antibodies. An enhanced chemiluminescence reagent (Millipore, USA) was used to detect the membranes using a GBOX‐CHEMI‐XT4 detection system (Syngene, UK).

### 4.22 Tissue immunofluorescence staining

Sections of brain tissues were embedded in paraffin and heated at *p*H 6 to retrieve antigens. Primary antibodies against Nrf2 (1:100) and HO‐1 (1:100) were respectively incubated with the tissues overnight at 4°C. Following washing, the Nrf2 and HO‐1 stained slides were incubated for 1 h at room temperature with Alexa Fluor 488‐ and Alexa Fluor 594‐conjugated secondary antibodies (1:200, Thermo Scientific). Slides were stained with DAPI and examined under the FluoView1000 (Olympus, Japan) confocal microscope.

### 4.23 Statistical analysis

The mean and standard deviation (SD) were used to present quantitative data. Student's *t*‐test and one‐way ANOVA were used for statistical analysis. *, **, and *** indicate *p* < 0.05, *p* < 0.01, and *p* < 0.001, respectively.

## CONFLICT OF INTEREST

Weibo Cai is a member of the *Exploration* editorial board; a scientific advisor, stockholder, and grantee of Focus‐X Therapeutics, Inc.; a consultant and grantee of Actithera, Inc.; a consultant of Rad Source Technologies, Inc.; a scientific advisor of Portrai, Inc.; and a scientific advisor and stockholder rTR Technovation Corporation. The other authors declare no conflict of interest.

## ETHICS STATEMENT

All procedures associated with animals were performed according to the National Institutes of Health Guide for the Care and Use of Laboratory Animals while the ethics were approved by the Institutional Animal Care and Use Committee of the Peking University First Hospital (No. 201822).

## Data Availability

All data related to this study are present in the article. Any other data associated with this work are available from the corresponding authors upon request.
